# The effect of OsteoStrong compared to dynamic multicomponent exercise on bone strength in older women: the BONEMORE non-inferiority randomized controlled trial

**DOI:** 10.1007/s11657-026-01679-9

**Published:** 2026-02-26

**Authors:** Peter W. S. Lindberg, Kristin Moystad Michelet, Christina Kaijser Alin, Eva Andersson, Ann-Charlotte Grahn Kronhed, Per Magnusson, Sven Nyrén, Hans Ranch Lundin, Eva Toth-Pal, Maria Sääf, Helena Salminen

**Affiliations:** 1 Department of Neurobiology, Care Sciences and Society, Karolinska Institutet, Stockholm, 171 77 Sweden; 2 Department of Physical Activity and Health, Swedish School of Sport and Health Sciences, Stockholm, 114 33 Sweden; 3 Department of Molecular Medicine and Surgery, Karolinska Institutet, Stockholm, 171 77 Sweden; 4 Department of Health, Medicine and Caring Science, Linköping University, Linköping, 581 85 Sweden; 5 Department of Clinical Chemistry, Department of Biomedical and Clinical Sciences, Linköping University, Linköping, 581 85 Sweden; 6 Academic Healthcare Centre Stockholm, Stockholm, 113 65 Sweden

**Keywords:** Osteoporosis, Exercise, Bone mineral density, Bone material strength index, Bone biomarkers, Older women

## Abstract

***Summary*:**

The efficacy of OsteoStrong® (OS) on bone strength is limited, and how it compares to dynamic multicomponent exercise (DME) is unknown. In this randomized controlled trial, the effect of OS on bone material strength index (BMSi) in older women was non-inferior to that of DME. No significant interaction between treatment and time was observed in any measured outcomes, indicating no meaningful difference between the intervention groups. Although the OsteoStrong® intervention met the pre-specified non-inferiority margin compared to the DME group, the lack of efficacy of the DME intervention on BMSi limits the interpretation of this finding.

**Purpose:**

The aim of this study was to investigate whether the effect of using OS was non-inferior to DME for the primary outcome of BMSi in older women.

**Methods:**

Women aged 65–79 years with osteopenia or osteoporosis were randomized to 9 months of once-weekly OS (20 min) or twice-weekly DME (60 min/session). A total of 194 women (OS, 97; DME, 97), median age 70 years, participated in the trial. The primary outcome was BMSi measured with impact microindentation at the tibia. A pre-specified non-inferiority margin of BMSi −5.2 was applied to determine whether the intervention’s efficacy was not clinically worse than the comparator within an acceptable threshold. The secondary outcomes were bone mineral density (BMD) and bone markers (S-CTX, S-P1NP, S-BALP, and S-sclerostin).

**Results:**

At the 9-month follow-up, there were no significant differences between the groups in any of the outcomes. The mean between‑group difference in BMSi was 1.16, with a 95% confidence interval ranging from − 1.51 to 3.82. Since the lower limit of the confidence interval (− 1.51) did not cross the predefined non‑inferiority margin of − 5.2, non‑inferiority was established. There was a significant increase in BMSi by 2.9% in the OS group (from 73.9 ± 9.5 to 76 ± 9.4, *p* = 0.025), and lumbar spine BMD by 0.8% in the DME group (from 0.866 g/cm^2^ ± 0.13 to 0.872 g/cm^2^ ± 0.13, *p* = 0.016). Changes in femoral neck BMD were not significant. There were no significant within- or between-group differences in any of the bone markers at 3 and 9 months.

**Conclusions:**

Based on these findings, the effect of OsteoStrong® on BMSi in older women was non-inferior to that of dynamic multicomponent exercise. No significant interaction between treatment and time was observed in any measured outcomes, indicating no meaningful difference between the intervention groups. Although the OsteoStrong® intervention met the pre-specified non-inferiority margin compared to the DME group, the lack of efficacy of the DME intervention on BMSi limits the interpretation of this finding.

**Supplementary information:**

The online version contains supplementary material available at 10.1007/s11657-026-01679-9.

## Introduction

Exercise as part of the management of osteoporosis is strongly recommended in international guidelines [1, 2]. Exercise has been shown to improve or maintain bone mineral density (BMD) [3, 4]. Although the effect of exercise on BMD has been studied quite extensively, its effect on other properties of bone strength, such as bone quality and bone markers, remains less understood. A measurement of bone quality is the bone material strength index (BMSi), which is measured in the tibia using a handheld device. The BMSi has been shown to be able to discriminate between fracture cases and controls, as well as being independent of BMD [5–7]. Some studies in dicate that a low BMSi correlates with a higher risk of fracture [5, 8, 9], but there have also been contradictory findings [10]. To our knowledge, only two clinical trials have investigated the effect of exercise on BMSi, with one trial showing an increase in jumping on one leg, and the other trial showing non-significant changes in multicomponent exercise [11, 12]. Bone markers have been shown to be associated with BMD [13, 14], and changes in bone markers occur more rapidly than changes in BMD when using bone-specific drug therapy [15]. Current data indicate that exercise induces both acute and medium-term effects on bone markers [16, 17]. However, the response to exercise seems to depend on the type of exercise modality, intensity, age, and sex [16, 17]. Although numerous studies have examined the relationship between bone markers and aerobic exercise [16, 17], the effects of resistance exercise on bone markers in older women remain underexplored. To our knowledge, no clinical trials have investigated the impact of isometric exercise on either BMSi or bone markers.

Most research on exercise and bone strength has focused almost exclusively on dynamic exercises (i.e., muscle contraction with visible joint movement). In contrast, studies investigating isometric exercise (i.e., muscle contraction without visible joint movement) targeting bone strength are very limited. Isometric exercise has been shown to have several health benefits similar to dynamic exercise, such as lowering blood pressure, improving pain and function in tendinopathy, and increasing muscle strength [18–20]. While physical loading is a well-established stimulus for bone formation [21], the specific effects of isometric exercise on bone strength remains poorly understood. In recent years, OsteoStrong®, a system utilizing machine-based isometric loading, has emerged as a widely marketed intervention, proposing that just one brief weekly session may be sufficient to strengthen bone. However, robust clinical evidence supporting these claims remains limited, warranting further investigation [22]. The primary aim of this study was to investigate whether the effect of OsteoStrong® is non-inferior to dynamic multicomponent exercise for the primary outcome of BMSi in older women. The secondary aim was to compare the effect of OsteoStrong® and dynamic multicomponent exercise on BMD and bone markers.

## Methods

### Study design and research ethics

This study was a two-arm randomized controlled trial conducted in Stockholm, Sweden. The eligible participants were randomized to 9 months of OsteoStrong® (OS) or dynamic multicomponent exercise (DME). The subject enrollment commenced in January 2022 and was completed in May 2023. The study was approved by the Swedish Ethical Review Authority (Dnr: 2020–04359). Written informed consent was obtained from all participants. The study was conducted according to the World Medical Association Declaration of Helsinki. All participants received written and oral information prior to study enrollment, and written consent was obtained from each study participant. The study protocol was registered at ClinicalTrials.gov (ID: NCT05721014).

### Rationale for non-inferiority design

This study employs a non-inferiority design to evaluate whether OS is not unacceptably less effective than DME in improving bone strength among older women. DME is an intervention based on current recommendations on physical activity for individuals with osteoporosis [2, 23]. It incorporates several different components, including strength- and balance training, as well as some weight-bearing impact training. Multicomponent exercise has been shown to be effective in maintaining or improving BMD in older women with osteoporosis [4, 24–26–27], reducing the risk of falls [28], muscle strength, balance, quality of life and functional outcomes [24–26, 29, 30]. A non-inferiority approach allows for the direct comparison of OS to this active control group, aiming to examine if OS could serve as a viable alternative exercise modality for individuals with osteoporosis. This approach might also consider potential advantages of OS, such as reduced time commitment and greater accessibility for individuals who have difficulties in participating in multicomponent exercise. Instead of aiming to show that OS might be superior, the study seeks to examine whether it retains sufficient clinical benefit within an acceptable margin of difference.

### Participants

Postmenopausal women between 65 and 79 years of age were recruited between September 2021 and June 2022 via newspapers ads, a women’s organization, and Facebook. Potential participants were screened for eligibility. The inclusion and exclusion criteria can be found in Table 1. The participants began their exercise program at six different points in time, depending on when they were recruited.

**Table 1:** Inclusion and exclusion criteria

Inclusion criteria	
• A *T*-score ≤ −1.0 at the hip and/or spine • Fully vaccinated against COVID-19 • Available for the trial for a period of 9 months • Either not receiving bone-specific treatment or undergoing such treatment continuously for at least 1 year prior to enrollment	
Exclusion criteria	
• Ongoing treatment with bone-specific drugs with an onset within the last year, due to the likelihood of not achieving a stable treatment effect; or previous drug treatment that was terminated within the last 5 years, due to the potential lingering effects of the medication • Ongoing bone-specific drugs that had been delayed for more than 8 months for denosumab and 18 months for bisphosphonates • Ongoing treatment with oral corticosteroids (≥ 5 mg prednisolone) • Vertebral fractures that were (a) diagnosed within the past 3 months, (b) not assessed by a physician and deemed suitable for study participation, or (c) not previously treated with bone-specific drugs • Bilateral hip replacement • Symptomatic disc herniation, inguinal herniation or umbilical herniation • Untreated hypertension • Other diseases that could affect the results of the study or participation (e.g., malignant diseases, secondary osteoporosis, muscle dystrophy) • Conditions that could hinder the impact microindentation test (large edema, skin infection, allergic to local anesthetics) • Ongoing or previous training at OsteoStrong® within the last year	

### Randomization and blinding

We used a stratified process to randomize eligible participants at the end of baseline testing, stratified by the presence or absence of bone-specific drug therapy. A computer-based randomization generator created the block randomization sequence, with block sizes varying between 4 and 6, generated in random order. These sequences were then assembled into envelopes by a researcher who was not involved in participant assessments. The researchers who allocated the participants were blinded to group assignments during the baseline assessment. All baseline measurements were conducted by blinded researchers. Blinding was not maintained at follow-up in order to preserve the standard assessment procedures and due to practical limitations and resource constraints. At follow-up, the bone marker analyses, DXA, and VFA assessments were blinded. It was not possible to blind the participants or the training instructors delivering the interventions.

### Interventions

#### Treatment arm A: OsteoStrong®

The OS intervention was carried out at the OS center in Solna, Sweden, once a week. It was conducted according to the OS concept, which includes high-intensity isometric axial bone loading using four different machines (Spectrum™); “upper growth trigger” (chest press), “lower growth trigger” (leg press), “core growth trigger” (pull-down), and “postural growth trigger” (deadlift). The exercise is referred to as “isometric” because, by definition, the muscle-tendon unit maintains a constant length during muscle contraction, even though the external load may vary [20]. The training also included 3–5 min of light warm-up and balance exercises (one-leg standing) on vibration platforms (Power Plate™). The duration of the short-term vibration used by the participants varied due to individual abilities and preferences. The frequency used on the vibration platforms was 30–35 Hz with an amplitude of 1–2 mm. At the start of the intervention, all participants received a 60-min introduction according to the OS concept. The participants performed the OS using machines by applying isometric force, pressing or pulling the handles and platform for approximately 15–20 s per machine. Based on the OS concept, the goal was to reach a force of 2.5 times their body weight on the upper and postural growth triggers (GT), 1.5 times their body weight on the core growth trigger, and 4.2 times their body weight on the lower growth trigger [31, 32]. This was monitored by the training instructor using the visible display on each machine, which showed the amount of force produced by each. This allowed the participants to receive direct feedback on their performance from the display. The total time allocated for the exercise program was 20 min. The exercises were individually adapted, with machine settings adjusted to participants’ height and instructional guidance modified according to each individual’s physical condition. Exercise intensity was progressively increased over the course of the intervention.

#### Treatment arm B: dynamic multicomponent exercise

The DME was performed at the Swedish School of Sport and Health Sciences in Stockholm twice per week. The DME was based on current recommendations on physical exercise for people with osteoporosis, including strength and balance training, as well as some weight-bearing exercises [23]. The program was divided into two training sessions (60 min each) led by an experienced training instructor. The first session was a circuit exercise program which included ten stations with different exercises for the whole body using some equipment (for the full list of exercises, see Appendix A), as well as a joint warm-up and cool-down exercises. No external load was used in the exercises, only the participants’ body weight. The duration for each station was 40 s and an interim rest for 20 s. The participants were instructed to do as many repetitions as possible on a level that was challenging, yet manageable. If unable to attend the group training session, they had the option to complete the workout as a home exercise program. The participants received the written exercise program, as well as a video of the exercises. The second session was performed once a week in smaller groups in a gym using strength training machines and other equipment. The session included eight exercises with a focus on improving muscle strength and balance (leg press, leg curl, latissimus pull-down, seated row, back extension, chest press, hip abduction, balance exercises), as well as warm-up and cool-down exercises. The participants were instructed to perform strengthening exercises with a weight load that allowed a maximum of 8–10 repetitions (corresponding to 75–80% of their one-repetition maximum) before reaching muscle failure. Each exercise was performed in three sets. The exercises were individually adjusted and progressed over time. The participants were given a logbook to log their strength training progress (i.e., the number of repetitions, sets and weights used per machine).

#### Safety and compliance

Both exercise interventions were supervised by experienced training instructors. The exercises were adapted to the participants’ functional ability and health status. The participants were recommended to (i) start slowly and learn the exercise before increasing the intensity and weight load to minimize the risk of injuries, (ii) disclose any health conditions for consideration by the training instructors, and (iii) report any injuries or adverse effects of the exercise. The training instructors were responsible for registering the participants’ attendance. The supervised training sessions helped to ensure exercise fidelity and adherence. The participants were instructed not to change their physical activity habits, apart from the interventions during the study period, until they had completed the 9-month follow-up visit. However, they were allowed to continue any physical exercise that was already ongoing prior to their enrollment in the trial.

#### Data collection

The participants were required to attend four visits to complete all study measurements, including body composition assessments, questionnaires, and bone strength evaluations (impact microindentation test, dual-energy X-ray absorptiometry, and bone marker analysis via blood tests). All data, except for the DXA and bone marker data, were collected at Karolinska Institutet (Stockholm, Sweden). Follow-ups were conducted at 3 and 9 months, respectively. However, the 3-month follow-up only included blood tests and questions about the participants’ current health and study status. Data collection started in October 2021 and ended in July 2023.

### Outcome measures

#### Primary outcome

##### Bone material strength index

BMSi was chosen as the primary outcome of this study due to its potential as a measure of bone quality, contributing to the assessment of fracture risk and providing valuable insights in this area. An additional reason was that changes in BMSi in response to physical loading have been observed within just a few months, potentially allowing for a faster response to physical exercise than with BMD [11]. Given that most individuals who experience a fragility fracture do not meet the diagnostic threshold for osteoporosis (*T*-score ≤ −2.5 SD) and considering that BMD accounts for only about 60–70% of the variability in bone strength, research on other aspects of bone strength, such as measures of BMSi, is essential [33–35].

The BMSi was measured with impact microindentation (IMI) using the handheld OsteoProbe® device (Active Life Scientific, Santa Barbara, CA, USA). IMI is a minimally invasive in vivo test performed on the mid-portion of the tibia to test bone fracture resistance and thus, bone quality. Before the test can be performed, local anesthesia is used in the test area. The IMI was conducted on the same mid-portion area of the tibia at both baseline and follow-up, which was determined by measuring the distance between the medial tibial malleolus and the apex of the patella. An OsteoProbe® needle was inserted through the skin and applied perpendicular to the tibial bone surface. The individual performing the test pushes down the device and, at a force of 10 Newton (N), triggers a mechanism, which makes the device deliver an impact of 40 N in less than a millisecond. This causes a small microfracture in the size of 375 μm. The vertical distance created by the cavitation from the indentation is called the indentation distance increase (IDI). In one IMI test, at least eight indentations are performed, with approximately 2 mm between the indentations. The test result remains concealed from the operator until all eight indentations have been completed. To generate the BMSi, the IDI is compared to the IDI of the IMI performed on a polymethylmethacrylate plastic calibration phantom, which acts as a reference and is equal to a BMSi value of 100.

The BMSi measurement was performed twice, at baseline and directly after the intervention ended. Three physicians performed the IMI. They had received the same training in the procedure with the OsteoProbe® device and were blinded at baseline, but not at follow-up due to logistical reasons. In a previous study that used the same procedure, the intra-observer coefficient of variation (CV) was 3.2%, and the inter-observer CV was 5.2% [11].

#### Secondary outcomes

##### Bone mineral density (BMD)

Measurements of areal BMD (g/cm^2^) were performed at the lumbar spine (L1–L4) and hips (femoral neck, greater trochanter, and total hip), with dual-energy x-ray absorptiometry (DXA) (Fuji, FDX Visionary-DR, Solna, Sweden; software v. 5.0.2.0) at baseline and directly after the intervention ended. Hips with hip replacement were excluded from the DXA measurements. After each DXA examination, the images were reviewed by a radiologist with over 30 years of experience in radiology and more than 20 years of experience in DXA. In cases where image quality issues were identified, the participant was recalled for reassessment. The same radiologic technologist performed all the DXA scans. The CV for the DXA equipment, measured against a phantom during the study period, was 0.38%. All DXA scans were conducted at the Bone Monitoring Clinic, Solna, Sweden.

##### Vertebral fracture assessment (VFA)

3The VFA was performed alongside the DXA scan to identify potential vertebral fractures. In this study, the VFA served two purposes: (a) as part of the eligibility screening during enrollment, ensuring the exclusion of women with newly identified vertebral fractures that were untreated or unassessed, and (b) as a safety measurement to compare the VFA findings at baseline and the 9-month follow-up. The VFA was performed with the person examined laying on the side, preferably with the right side down, with flexed legs and arms. Supporting cushions were placed behind the back and between the bent knees to give more stability and to avoid twists in the position. The VFA imaging was performed by the same radiologic technologist, who was blinded to participant allocation. The VFA images were assessed by a qualified radiologist (SN), who used Genant’s classification system (grade 0–3) when assessing the spine images [36]. The radiologist was blinded to participant allocation.

##### Bone markers

Blood samples were drawn in the morning after an overnight fast at baseline and follow-up after 3 and 9 months. Aliquots were stored at −80 °C until the analyses were performed. All assays were performed at the Department of Clinical Chemistry (Swedac accredited no. 1342), Linköping University Hospital, Sweden, and all samples were analyzed in duplicates with kits from the same batch.

Serum type I procollagen intact N-terminal propeptide (PINP) was assessed with the UniQ radioimmunoassay (Aidian Oy, Espoo, Finland), with the following assay performance: analytical range 5–250 µg/L, intra-assay CV < 5%, and inter-assay CV < 6%. Serum BALP was measured by the MicroVue™ BAP enzyme-linked immunosorbent assay (ELISA) (Quidel Corp., San Diego, CA), with the following assay performance: analytical range 0.7–140 U/L, intra-assay CV < 6%, and inter-assay CV < 8%. Serum C-terminal telopeptide cross-links of collagen type I (CTX) were measured in plasma samples by the CrossLaps® ELISA (Immunodiagnostic Systems Holdings PLC, Boldon, UK), with the following assay performance: analytical range 20–3380 ng/L, intra-assay CV < 6%, and inter-assay CV < 10%. Serum sclerostin was measured by quantitative ELISA (Biomedica, Vienna, Austria), with the following assay performance: analytical range 3.2–240 pmol/L, intra-assay CV < 7%, and inter-assay CV < 10%.

##### Statistical analysis

A non-inferiority approach with a pre-specified non-inferiority margin of −5.2 was used, which was the mean difference in BMSi between the intervention group and the control group in another study [11]. This value was also used for the power calculation in the present study. A total of 112 participants were required to achieve a minimum of 90% statistical power to detect between-group differences of 5.2 ± 9.0 in BMSi, based on a two-sided alpha level of 0.05. Although a two-sided alpha was used for the calculation, the non-inferiority assessment focused on whether the lower bound of the confidence interval for the new treatment’s BMSi exceeded the non-inferiority margin. Our goal was to enroll at least 160 participants to allow for dropouts. Both intention-to-treat (ITT) and per-protocol (PP) analyses were performed for all outcome measures. Participants with an attendance rate of at least 70% were included in the PP analysis.

Data were presented as mean (M) ± standard deviations (SD) or median (Md) (interquartile range, IQR) for continuous variables, and number (*n*) and percentages (%) for categorical variables. All tests were two-sided and a significance level of *ρ* < 0.05 was considered significant. A linear mixed model (LMM) was used to analyze the within- and between-group differences in BMSi and BMD at baseline and 9 months, and for bone markers at baseline, 3 months, and 9 months; with the fixed effects being the type of exercise (treat) and time (months), and the random effect being the participant ID. The interaction treat#months was included in the model to assess whether the trajectory of the outcome differed between treatment groups over time. Model parameters were estimated using restricted maximum likelihood (REML). Transformation and back-transformation according to the Ladder of Powers were used when the data were skewed, in order to make the model meet the assumptions. We used cube transformation on BMSi, and log transformation on PINP, BALP, CTX, and sclerostin. The random effect was the participant ID.

Individual and mean changes in BMSi and BMD were plotted alongside the least significant change (LSC), defined as the minimal difference between two consecutive measurements that can be confidently interpreted as a true biological variation rather than an artifact of measurement error or variability. LSC was calculated using the formula: LSC = Precision Error × 2.77. The LSC for BMSi, calculated as 8.86%, was derived from an intra-observer coefficient of variation (CV) of 3.2% reported in a previous study [11]. Corresponding values for DXA were determined using the radiologic technologist’s LSC thresholds—0.059 g/cm^2^ for lumbar spine BMD, 0.061 g/cm^2^ and 0.058 g/cm^2^ for right and left femoral neck BMD, respectively—based on repeated measurements in 30 patients.

To explore potential effect modification, we conducted post hoc linear regression analyses for each outcome. Two interaction terms were tested: (a) the interaction between ongoing bone-specific drug therapy at baseline and the intervention, and (b) the interaction between baseline prevalent fracture status and the intervention. Each model included the main effects and the respective interaction term. The subgroup analyses comparing were performed using the same LMM approach as in the main analysis. All outcomes were adjusted for age. All statistical analyses were performed using STATA 18 (StataCorp. 2023. Stata Statistical Software: Release 18. College Station, TX: StataCorp LLC).

## Results

### Enrollment and allocation

**Fig. 1:**
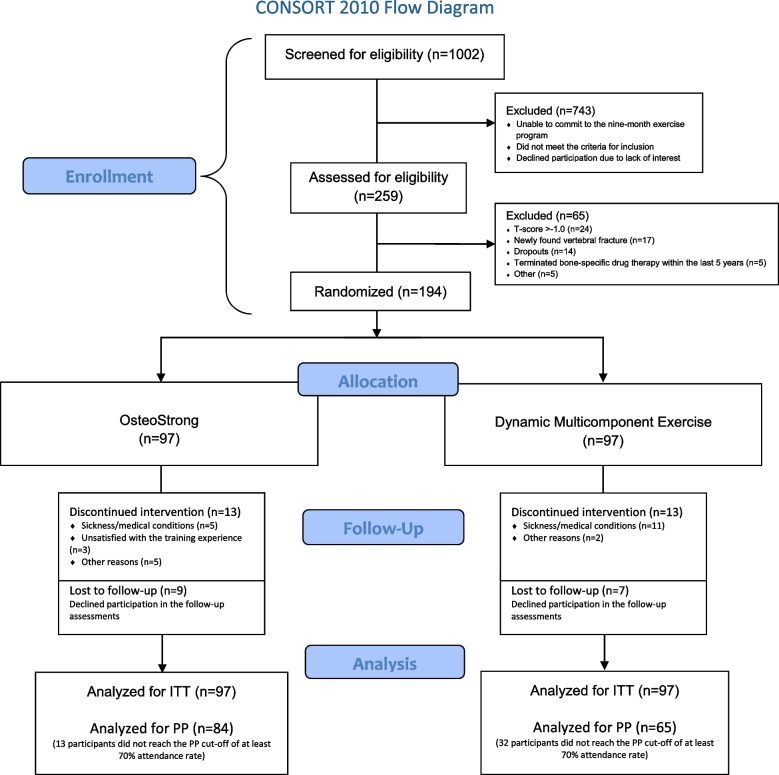
CONSORT study flow diagram showing participant enrollment, allocation, follow-up and analysis. ITT, intention-to-treat; PP, per-protocol

A total of 194 women, with a median age of 70 years, ranging from 65 to 79 years, were randomized to either OS (*n* = 97) or DME (*n* = 97). Of the 194 participants, 168 (86.6%) completed the trial, while 26 (13.4%) dropped out, evenly divided between the two groups (13 per group). In total, 178 women (91.7%) participated in the 9-month follow-up. See Fig. 1 for the full enrollment and allocation flowchart. The ITT analysis included 194 and the PP analysis included 149 participants. Of those not eligible, common reasons for exclusion were unable to attend the exercise sessions (location and time), recent onset or cessation of bone-specific drug therapy, *T*-score > −1.0, and untreated vertebral fractures. At baseline, a total of eight participants (4.2%) were using hormone replacement therapy.

### Baseline characteristics

There were no statistically significant differences between the groups in any of the baseline characteristics (Table 2). Only three women (1.6%) were classified as osteoporotic with a *T*-score ≤ −2.5, and 15 (7.7%) had a *T*-score ≤ −2.0 in the femoral neck (NHANES III) [37]. Based on lumbar spine BMD, there were 51 (26%) women with a *T*-score of ≤ −2.5, and 77 (40%) with a *T*-score of ≤ −2.0. A total of 23 participants (OS = 11, DME = 12) had been receiving bone-specific medication continuously for at least 1 year prior to enrollment. For details on the distribution of specific drug treatments across groups, please refer to Supplementary Table 1. The median baseline BMSi was 75.9 (IQR, 70.2–79.8) in the OS group and 76.8 (IQR, 69.3–82.1) in the DME group—both lower than reference values for women aged 65–74 years (80.6; IQR, 74.5–84.6) and 75 or older (79; IQR, 73.6–85) [38]. At baseline, 39% of participants had BMSi values below the lower quartile cutoff (< 73.6), while 8% exceeded the upper quartile cutoff (> 85). By the 9-month follow-up, these proportions shifted to 32% below the lower threshold and 20% above the upper threshold.

**Table 2:** Baseline characteristics of the participants (*n* = 194)

Parameter	OS (*n* = 97)	DME (*n* = 97)
Age (median years, IQR)	70 (67–75)	70 (67–75)
Weight (mean kg, SD)	64 ± 9	64 ± 9
Height (mean cm, SD)	163 ± 6	162 ± 6
BMI (mean kg/m^2^, SD)	24.2 ± 3.4	24.1 ± 3.4
Bone-specific drugs^a^ (*n*, %)	11 (11%)	12 (12%)
Previous fracture (*n*, %)	48 (49%)	51 (54%)
FRAX score MOF% (median, IQR)	12 (9.1–19)	13 (8.75–17)
FRAX score hip fracture % (median, IQR)	1.9 (1–4)	1.9 (0.9–3.9)
BMSi (mean, SD)	73.9 ± 9.46	74.7 ± 9.68
BMD LS Total (mean g/cm^2^, SD)	0.874 ± 0.130	0.866 ± 0.127
BMD FN Right (mean, SD)	0.756 ± 0.101	0.763 ± 0.094
BMD FN Left (mean, SD)	0.754 ± 0.091	0.758 ± 0.084
*T*-score LS Total (mean, SD)	−1.55 ± 1.24	−1.68 ± 1.15
*T*-score FN Right (mean, SD)	−0.848 ± 0.846	−0.791 ± 0.786
*T*-score FN Left (mean, SD)	−0.870 ± 0.762	−0.830 ± 0.708
P1NP (median, IQR)	48.9 (38–63)	47.7 (35–61)
BALP (median, IQR)	20.4 (15–24)	19.1 (16–24)
CTX (median, IQR)	370 (258–504)	370 (239–513)
Sclerostin (median, IQR)	26.3 (20–32)	25.8 (21–33)
Meeting WHO’s recommendation of 150–300 min of weekly physical activity (*n*)^b,c^	87 (90%)	81 (84%)
Weekly physical exercise levels (minutes)^b^	48.9 ± 45.3	42.1 ± 43.7
Other weekly physical activity levels (minutes)^b^	206.9 ± 85.8	191.3 ± 97.9

*BMI* body mass index, *MOF* major osteoporotic fracture, *FRAX* fracture risk assessment tool, *BMSi* bone material strength index, *BMD* bone mineral density, *LS* lumbar spine, *FN* femoral neck, *P1NP* N-terminal propeptide of type-1 procollagen, *BALP* bone alkaline phosphatase, *CTX* C-terminal telopeptide of type I collagen

^a^Ongoing treatment with bisphosphonates (*n* = 23) for at least a year: alendronate (48%), zoledronic acid (35%), denosumab (17%)

^b^Self-reported physical activity with at least moderate intensity

^c^Total number of participants meeting the recommendation of 150–300 min of weekly physical activity (moderate intensity) = 168 (87%)

### Effects on BMSi

Changes in BMSi over the course of the trial are presented in Table 3. The ITT analysis, adjusted for age, revealed no significant difference between the groups (LMM post hoc comparison, *ꭓ*^2^ = 0.02, *p* = 0.881). The mean between‑group difference in BMSi was 1.16, with a 95% confidence interval ranging from − 1.51 to 3.82. Since the lower limit of the confidence interval (− 1.51) did not cross the predefined non‑inferiority margin of − 5.2, non‑inferiority was established. The within-group analysis showed a significant increase in BMSi in the OS group by 2.9% (LMM post hoc comparison, *ꭓ*^2^ = 5.02, *p* = 0.025), whereas the BMSi did not increase over time in the DME group (*ꭓ*^2^ = 1.30, *p* = 0.254). The LMM revealed a significant main effect of time (*p* = 0.025), indicating that the outcome improved over the study period. Additionally, the interaction term between treatment and time was not significant (*p* = 0.431), suggesting that the rate of improvement over time did not differ between the treatment groups. Individual and mean changes in BMSi relative to the LSC are presented in Supplementary Figure S1, with the majority of the values remaining below the LSC threshold. The PP analysis (Supplementary Table S2) yielded similar results to the ITT analysis, with no significant difference between the groups. However, there was a change in BMSi of 2.8% in the DME group, showing a trend that approached significance (DME, *ꭓ*^2^ = 3.46, *p* = 0.063). Ninety percent of the IMI tests were conducted by the same physician on the same participant at baseline and follow-up.

**Table 3:** Age-adjusted intention-to-treat analysis within-group change, and between-group difference in BMSi, and BMD, displayed per group and time points, presented in mean ± SD. The *p*-values were obtained from the linear mixed model. *p*-value ≤ 0.05 in bold

	OsteoStrong® (*n* = 97)			Within-group change	Dynamic multicomponent exercise (*n* = 97)			Within-group change	Between-group difference
	Baseline Mean ± SD	9 months Mean ± SD	% change	*p*-value	Baseline Mean ± SD	9 months Mean ± SD	% change	*p*-value	*p*-value
BMSi	73.9 ± 9.5	76 ± 9.4	+ 2.9%	**0.025**	74.7 ± 9.7	75.9 ± 8.8	+ 1.6%	0.257	0.881
BMD LS Total (g/cm^2^)	0.874 ± 0.131	0.871 ± 0.130	−0.3%	0.136	0.866 ± 0.127	0.873 ± 0.132	+ 0.8%	**0.016**	0.734
BMD FN Right (g/cm^2^)	0.756 ± 0.102	0.752 ± 0.097	−0.5%	0.771	0.763 ± 0.094	0.763 ± 0.095	−0.02%	0.653	0.765
BMD FN Left (g/cm^2^)	0.754 ± 0.091	0.758 ± 0.091	+ 0.6%	0.076	0.758 ± 0.085	0.762 ± 0.091	+ 0.4%	0.471	0.910

*BMSi* bone material strength index, *BMD* bone mineral density, *FN* femoral neck, *LS* lumbar spine, *SD* standard deviation

### Effects on BMD

Changes in BMD over the course of the intervention are presented in Table 3. The ITT analysis, adjusted for ages, revealed no significant difference between the groups (LMM post hoc comparisons, *ꭓ*^2^ = 0.11, *p* = 0.739). The within-group analysis showed a significant increase in lumbar spine BMD in the DME group of 0.8% (LMM post hoc comparison, *ꭓ*^2^ = 5.78, *p* = 0.016), but not in the OS group (*ꭓ*^2^ = 2.22, *p* = 0.136). This change remained significant for the DME group after removing individuals with vertebral fractures at follow-up. There were no significant changes in right or left femoral neck BMD in any group or any between-group differences. Individual and mean changes in lumbar spine and femoral neck BMD relative to the LSC are shown in Supplementary Figure S1, with very few values exceeding the LSC threshold. The PP analysis (Supplementary Table S2) showed similar results as the ITT analysis, except that the change in lumbar spine BMD in the DME group was not significant (*ꭓ*^2^ = 0.59, *p* = 0.444). Additionally, the change in lumbar spine BMD in the OS group ranged from −0.2 to + 0.5% (*ꭓ*^2^ = 2.45, *p* = 0.117), but did not reach statistical significance.

### Effects on bone markers

Changes in bone markers over the course of the intervention are presented in Table 4. In the ITT analysis, adjusted for age, there were no significant changes in any of the bone markers in either group at any time point, and no between-group differences were observed. The PP analysis (Supplementary Table S3) yielded results that were consistent with the intention-to-treat analysis, with no significant differences observed between the two approaches.

**Table 4:** Age-adjusted intention-to-treat analysis within-group change, and between-group difference in bone markers displayed per group and time points, presented in median (IQR) and percentage change (compared to baseline). The *p*-values were obtained from the linear mixed model. No significant differences were found within or between the groups in any of the bone markers at any time points

	OsteoStrong® (*n* = 97)			Dynamic multicomponent exercise (*n* = 97)			Between-group difference
	Baseline Median (IQR)	3 months Median (IQR), %	9 months Median (IQR), %	Baseline Median (IQR)	3 months Median (IQR), %	9 months Median (IQR), %	*p*-value
PINP (µg/L)	49 (38–63)	45 (37–62); −8%	49 (37–61); + 0.8%	48 (35–61)	48 (37–59); + 0.01%	49 (37–59); + 1.8%	3 months, 0.941 9 months, 0.830
BALP (U/L)	20.4 (14–24)	20.5 (16–26); + 0.6%	20 (15–26); −2.1%	19.1 (16–24)	19.3 (16–24); + 0.6%	19.8 (15–24); + 3.4%	3 months, 0.399 9 months, 0.702
CTX (ng/L)	370 (258–504)	375 (210–505); + 1.4%	367 (253–525); −0.8%	370 (239–513)	365 (241–496); −1.5%	361 (241–474); −2.6%	3 months, 0.930 9 months, 0.890
Sclerostin (pmol/L)	26.343 (20–32)	25.5825 (21–32); −2.9%	26.62475 (22–31); + 1.1%	26 (21–33)	25 (21–31); −1.5%	28 (24–36); + 7.4%	3 months, 0.396 9 months, 0.160

*BALP* bone alkaline phosphatase, *CTX* C-terminal telopeptide of type I collagen, *P1NP* N-terminal propeptide of type-1 procollagen, *IQR* interquartile range

### Post hoc regression analyses

Age-adjusted analyses revealed no significant differences compared to the unadjusted analyses across any of the outcomes. Regression analyses examining potential effect modifiers—including (a) the interaction between ongoing bone-specific drug therapy and the intervention, and (b) the interaction between baseline prevalent fracture status and the intervention—revealed no statistically significant effects on any of the studied outcomes. These results suggest that neither concurrent bone-specific medication nor prior fracture history meaningfully influenced the intervention’s primary effects on the outcomes measured.

### Sub-analyses

We performed an ITT sub-analysis, using LMM, examining the effect of OS and DME on bone strength parameters in participants with and without ongoing bone-specific drug treatment (Appendix B). In the OS subgroup without bone-specific drug treatment, the within-group increase in BMSi rose from 2.9 to 3.2% (*p* = 0.023). Meanwhile, in the DME group, the increase in lumbar spine BMD rose from 0.8 to 0.9% (*p* = 0.048). In the OS subgroup with bone-specific drug treatment, there was a significant increase in PINP of 25.7% at 9 months, based on data from eight participants. No significant changes in BMSi or BMD were observed within any subgroups with bone-specific drug treatment. No significant interaction effects were observed between OS and DME regarding any measured outcomes of the sub-analyses with or without bone-specific drug treatment. Although, there was one exception observed for the left femoral neck BMD with higher values for OS compared to DME in the analysis among those with bone-specific drug treatment. However, the number of participants was here very low (OS = 11, DME = 12).

We also performed a PP sub-analysis assessing the effect of the OS intervention alone, comparing subgroups who reached the trigger levels (or beyond) a 100% of the sessions in the lower and postural GT and those who did not (Appendix C). The lower and postural GT were selected for analysis because they were considered to place the greatest load on the hips and spine, which were measured with DXA, and on the tibia, which was measured with IMI. The cutoff at 100% for the trigger levels was chosen because OS defines this threshold as the point participants must reach to elicit an osteogenic response in the bone. The sub-analysis revealed no significant changes in the subgroup that reached the trigger levels in 100% of the sessions. In contrast, the other subgroup, which achieved mean trigger levels in 80% and 85% of the sessions, respectively, demonstrated significant increases in BMSi and femoral neck BMD in the lower GT sub-analysis, as well as a significant increase in BMSi in the postural GT sub-analysis. At follow-up, a significant difference in lumbar spine BMD emerged between the subgroups. Excluding participants with bone-specific drug treatment did not diminish these effects.

### Vertebral fractures

At baseline, 21 women had vertebral fractures (OS = 10, DME = 11; *χ*^2^ *p* = 0.817). At the 9-month follow-up, new vertebral fractures were detected in four individuals (OS = 3, DME = 1). Of these, two fractures (one in each group) were confirmed as having resulted from accidents unrelated to the intervention, prompting the affected participants to withdraw from further participation in the intervention. Both individuals continued to receive care for their conditions; one was already on bone-specific medication, while the other was in the process of initiating such treatment. For the remaining two cases (OS = 2, DME = 0), the cause of the vertebral fractures could not be determined and the affected participants reported no symptoms during the active intervention period. They were informed of the findings and referred to primary healthcare for continued management.

### Safety and adherence

Of the 194 women who were randomized, a total of 168 completed the 9-month exercise intervention, and 26 (OS = 13, DME = 13) dropped out. The main reasons for dropouts were injuries or sickness (OS = 5, DME = 11), dissatisfaction with the training (OS = 3, DME = 0), and other reasons (OS = 5, DME = 2). The overall mean attendance rate for the OS group was 94%, and for the DME group 81% (of which 3.5% were home exercise sessions), not including dropouts. In the OS group, the percentage of women who had reached or surpassed the trigger level for each training machine was 68, 87, 92, and 92 for the upper, lower, core, and postural growth trigger, respectively. There were a total of 32 reported adverse events (AEs), of which 18 occurred outside of the study. A total of 14 AEs (OS = 9, DME = 5; Fisher’s exact *p* = 0.406) were reported in relation to the exercise intervention. The most common AE was gradual onset of musculoskeletal pain (*n* = 10; OS = 8, DME = 2), followed by sudden onset of pain (*n* = 2; OS = 1, DME = 1), one fall (DME = 1), and one incident of dizziness (OS = 1). In five out of the 12 cases of musculoskeletal pain, the participants had a history of previous pain issues in that part of the body. One serious AE involved a woman in the DME group who fell off a balance board, resulting in a triquetral fracture. Regarding the microindentation test with OsteoProbe®, only one participant reported lingering, though gradually subsiding, pain after a single measurement. No other persistent pain or adverse events were reported in connection with the procedure.

## Discussion

### Summary of key findings

The aim of the BONEMORE trial was to determine the efficacy of OsteoStrong® compared to dynamic multicomponent exercise in improving bone strength in older women over a 9-month intervention. The non-inferiority hypothesis, that the OS would not be inferior to DME in the effect on BMSi, BMD, and bone markers in older women, was confirmed. Both exercise interventions produced similar results with no significant differences between the groups in any of the outcomes.

### Interpretation of the results and comparison with previous research

To our knowledge, only two clinical trials have investigated the efficacy of physical exercise on BMSi [11, 12]. One study evaluated a 3-month daily unilateral high-impact exercise (jumping on one leg) in 20 healthy women (mean age 55.6 years) [11]. The BMSi in the jumping leg increased from 73.4 to 76.8 (4.6%), while the control leg decreased from 76.6 to 74.8 (− 2.4%). Relative to the control leg, the BMSi of the jumping leg showed a net increase of 7% (*p* = 0.046). The increase in BMSi observed in their study was greater than in ours. The greater BMSi response observed in their study, compared to ours, may be attributable to the intervention design: their protocol incorporated high-frequency, high-impact unilateral jumps, a stimulus not replicated in the OS or DME programs, which relied on lower-impact or resistance-based exercises. Additionally, differences in sample size, population characteristics, and follow-up duration may have contributed to the observed discrepancy in outcomes.

Another study investigated whether a multicomponent exercise program could enhance bone properties in patients who had undergone bariatric surgery [12]. Eighty-four participants (80% women, mean age 42–47 years) were randomized to either a multicomponent exercise group or a control group that received standard care. After 11 months of training, they found no statistically significant differences in BMSi within or between the groups. Although their study focused on a younger population with different health profiles, the lack of significant improvement in BMSi aligns with our findings in the DME group. These results highlight the need for further research to identify which factors might enhance the responsiveness of bone material strength to exercise interventions. It should be noted that the participants in that study had undergone bariatric surgery, which is associated with significant changes in body composition and bone metabolism, potentially limiting the generalizability of their findings to our population of older women with osteopenia or osteoporosis.

Our study shows that BMSi might react positively to physical exercise. Although there was no significant difference between the groups, the increase in BMSi was significant within the OS but not in the DME group. A possible explanation for this could be due to the magnitude of the mechanical loading in the specific exercises. The bone must undergo a certain level of strain to stimulate the osteogenic effect [21]. The magnitude of the loading in OS was high, where the goal was to reach or surpass a load of 4.2 times the participant’s body weight on the lower GT machine (leg press), and 2.5 times on the postural GT machine (deadlift). The number of women in the OS group who reached and surpassed such trigger levels for these machines was 87% and 92%, respectively. Another possible explanation for the lack of increase in BMSi in the DME group is that, although the program included some impact exercises—such as jogging and skater jumps—the magnitude of the mechanical loading may not have been sufficient to elicit an osteogenic response in the tibia. Lastly, the studied population already had high levels of physical activity at baseline, which may have made it more challenging to achieve changes in the bone strength measures.

In relation to LSC, the majority of the individual changes in BMSi remained below the LSC threshold, although some exceeded it. For BMD values, only a few individual measurements surpassed the LSC threshold. Since more individuals exceeded the LSC threshold for BMSi than for BMD, these findings suggest a greater likelihood of achieving a true increase in BMSi from these interventions over 9 months compared to BMD. If this is the case, our results support the hypothesis that BMSi responds more rapidly to physical exercise than BMD. However, evidence regarding the clinical relevance of BMSi and the implications of changes in BMSi remain limited.

Although the OS was found to be non-inferior to DME in its effect on BMSi, it is important to note that in non-inferiority trials, intention-to-treat analysis tends to minimize differences between treatment groups, and thereby increases the likelihood of demonstrating non-inferiority.

Recently, two other clinical trials have examined the effect of OsteoStrong® on BMD in peri-/postmenopausal women [39, 40]. The first study was a Greek non-randomized controlled trial which included 147 participants (mean age range, 58–64 years) separated into two main groups: group A, which received OsteoStrong®, and group B, which served as the control group. Both groups were further subdivided based on the use of bone-specific drugs. In the OsteoStrong®-only group, lumbar spine BMD showed a modest increase from 0.815 at baseline to 0.821 (+ 0.7%) at 12 months, which reached statistical significance with a non-parametric test (0.019) but not significant after the Bonferroni adjustment (*p* = 0.91). No significant changes were observed in femoral neck BMD, total hip BMD, or trabecular bone score in the OsteoStrong®-only group. Furthermore, no significant changes were detected in PINP or CTX levels in any of the groups. The absence of significant changes in lumbar spine and femoral neck BMD, as well as the bone markers PINP and CTX, aligns with the findings of our study.

The second study was a single-arm pilot study from Australia, including 44 postmenopausal women aged 61.2 ± 5.5 years, who participated in 8 months of intervention with OsteoStrong® [40]. Consistent with our findings, no significant changes in BMD (total hip, femoral neck, and lumbar spine) were observed at the 8-month follow-up. Furthermore, sub-analyses on participants who reached the trigger levels in ≥ 26 sessions (75%) also did not show any benefits in bone measurements.

Previous research on physical exercise interventions for postmenopausal women suggests that moderate to high-intensity, dynamic multicomponent exercise programs incorporating resistance exercises may result in slight increases in BMD at the lumbar spine and hip, though there are some conflicting data [3, 4, 30, 41, 42]. A meta-analysis including postmenopausal women with a median age of 60 years suggests that high-intensity exercise might be the most effective way of increasing lumbar spine BMD [41]. In our study of older women with a median age of 70 years, a small but statistically significant increase (0.8%) in BMD was observed in the DME group at follow-up, whereas no such increase was seen in the OS group. Additionally, no changes in BMD were observed at the femoral neck in either group. Other studies have found increases in lumbar spine BMD of approximately 1–2% in postmenopausal women [42–44], although some have found no improvement at all [30, 45]. Furthermore, in contrast to our findings, several studies have reported significant increases in femoral neck BMD following exercise interventions that included resistance training [4, 30, 41], although consistent with our results, other studies found no significant changes [3, 46]. It is possible that the participants in our study did not achieve sufficient physical intensity or that the intervention period was not long enough to observe improved BMD. However, it is obvious that the women in our study already had high levels of physical activity at baseline. Furthermore, postmenopausal women typically experience an annual decrease in BMD [47]. Thus, maintaining BMD may be clinically valuable for this population.

Previous research on the effects of exercise on bone markers has been inconsistent, with some studies showing increases in bone formation markers (e.g., BALP, osteocalcin) [48–50], and reductions in resorption markers (e.g., CTX, NTX) [49, 51, 52], while other studies found no significant changes or decreases in these markers [49–53]. These inconsistent results may stem from several factors, including heterogeneity among studies, such as differences in study populations, interventions, follow-up durations, and the timing of blood sample collection relative to an acute exercise bout or resting values after a prolonged period of exercise. In our study, there were no significant changes in PINP, BALP, CTX or sclerostin at any time point within any group, nor were there significant differences between the groups. This could be because many participants were already engaging in some form of exercise at baseline, reducing the likelihood of changes in bone markers. This factor should also be considered when interpreting the BMSi and BMD findings. Additionally, the participants’ levels of physical activity during their leisure time, beyond the organized exercise sessions, may have influenced the results. Lastly, the absence of significant changes in bone markers may also reflect the relatively high intra-assay variability (CV ~ 6–10%) inherent in these biochemical measurements, which could obscure smaller but potentially meaningful biological responses.

Although the OS program included the use of vibration platforms, these were only used for up to 3–5 min per session for balance and warm-up exercises. This would amount to a total of 113–188 min based on the attendance rate of 94% (excluding the dropouts) during the 9-month intervention. This dose falls far below the 7000 min suggested by a systematic review and meta-analysis as potentially necessary to improve lumbar spine BMD via whole-body vibration training [54]. Moreover, another meta-analysis found no significant effect of whole-body vibration training on lumbar spine or femoral neck BMD [55]. Given these findings, the vibration component in our study was likely insufficient to meaningfully influence bone outcomes. Regarding any possible impact of the short-term vibration on BMSi, to our knowledge, the effect of whole-body vibration on BMSi in older women has never been investigated. However, it seems unlikely to have a significant impact due to the low exposure, although this highlights the need for further research on the topic.

In the sub-analysis comparing subgroups with and without bone-specific drug treatment, there was a significant 0.5% decrease in lumbar spine BMD in the OS group not receiving drug treatment, whereas no such change was observed in the DME group. This suggests that the OS intervention was not effective in improving lumbar spine BMD. In the second sub-analysis, comparing OS subgroups that reached the trigger levels in all training sessions with those that did not, significant improvements were observed only in the subgroup that did not reach the trigger levels in every session, but instead achieved a mean of 80% for lower GT and 85% for postural GT. This difference can neither be attributed to the inclusion of participants with bone-specific drug treatment, as excluding them did not reduce the effects, nor can it be explained by attendance rates, which were very similar between the groups. A plausible explanation may involve variations in baseline bone strength and the corresponding potential for improvement in these parameters.

### Strength and limitations

The strengths of this study include the large sample size, comprehensive evaluation of bone strength, high attendance rate, relatively low dropout rate, inclusion of VFA, and blinded outcome assessments of DXA, VFA, and bone markers. Additionally, all the DXA and VFA measurements were performed and assessed by the same persons, and 90% of the IMI were conducted by the same physicians. Lastly, this study was a randomized controlled trial, which inherently reduces biases and increases the reliability of the findings. The study has several limitations. First, the validity of BMSi remains uncertain, particularly its ability to assess fracture risk, as it is based on a limited number of cross-sectional and longitudinal studies. Additionally, the minimal clinically important difference for BMSi is unknown, making it unclear whether the specified non-inferiority margin of 5.2 in our study was appropriate. However, a key purpose of this study was to contribute knowledge about how physical exercise might affect bone quality, which BMSi is designed to measure, as BMD alone cannot fully explain fracture risk. Second, intra- and inter-observer CV measurements for BMSi were not conducted. This decision was based on ethical considerations, as repeating the invasive IMI test multiple times on participants was deemed inappropriate. However, the physicians adhered to a standardized testing protocol, and 90% of the measurements were performed by the same physician, which supports consistency in data collection. Third, we did not perform a power calculation for the secondary outcomes (BMD and bone markers) as our analyses were exploratory. This limits our ability to assess the reliability of the observed trends and draw definitive conclusions regarding the effects of the interventions on these parameters. Fourth, we did not perform repeated one-repetition maximum (1RM) tests to ensure exercise fidelity in the DME group. Regular 1RM testing could have resulted in more accurate monitoring of the participants’ strength levels, ensuring that the prescribed exercise intensities remained appropriately matched to the participants’ capabilities throughout the intervention. Lastly, the specific DME program included in this study has not been examined in previous trials concerning its effect on BMSi, which raises questions about its suitability for a non-inferiority design. However, the DME program was developed based on current exercise recommendations, incorporating strength and balance training, as well as weight-bearing and functional exercises that have been previously shown to positively influence BMD, physical strength, and balance.

### Clinical implications

Based solely on the study findings, no firm conclusions can be drawn regarding which exercise method should be recommended. Further research on OsteoStrong® is warranted to establish its efficacy in enhancing bone strength and to explore its potential benefits for other key outcomes in older women, such as muscle strength, balance, mobility, and self-reported health outcomes. Ultimately, clinical decision-making should align with established exercise guidelines while prioritizing individual patient needs, preferences, and safety. Regardless of the specific exercise methods, personalization and progressive adaptation are fundamental to enhance adherence while minimizing injury risk.

## Conclusion

This 9-month randomized controlled trial compared the effects of OS and DME on bone strength in older women. No significant interaction between treatment and time was observed in BMSi, BMD, and the measured bone markers (PINP, BALP, CTX, and sclerostin), indicating no meaningful difference between the intervention groups. OS was found to be non-inferior compared to DME with respect to BMSi. Within-group analyses showed modest increases in BMSi for OS and lumbar spine BMD for DME, but no changes in femoral neck BMD or bone markers were observed in any of the groups. Although the OsteoStrong® intervention met the pre-specified non-inferiority margin compared to the DME group, the lack of efficacy of the DME intervention on BMSi limits the interpretation of this finding. Given these limitations, future research should incorporate a placebo-controlled design to more definitively establish the efficacy of OS.

## Supplementary information

Below is the link to the electronic supplementary material.

ESM 1 (DOCX 16.6 KB)

ESM 2 (DOCX 32.2 KB)

ESM 3 (DOCX 36.1 KB)

ESM 4 (DOCX 481 KB)

ESM 5 (DOCX 16.3 KB)

ESM 6 (DOCX 44.4 KB)

## Data Availability

Data from this study are available upon reasonable request to the corresponding author after an assessment of confidentiality due to ethical and legal restrictions according to national legislation.
