# Correction: The effect of OsteoStrong compared to dynamic multicomponent exercise on bone strength in older women: the BONEMORE non-inferiority randomized controlled trial

**DOI:** 10.1007/s11657-026-01700-1

**Published:** 2026-04-23

**Authors:** Peter W. S. Lindberg, Kristin Moystad Michelet, Christina Kaijser Alin, Eva Andersson, Ann-Charlotte Grahn Kronhed, Per Magnusson, Sven Nyrén, Hans Ranch Lundin, Eva Toth-Pal, Maria Sääf, Helena Salminen

**Affiliations:** 1 Department of Neurobiology, Care Sciences and Society, Karolinska Institutet, 171 77 Stockholm, Sweden; 2 Department of Physical Activity and Health, Swedish School of Sport and Health Sciences, 114 33 Stockholm, Sweden; 3 Department of Molecular Medicine and Surgery, Karolinska Institutet, 171 77 Stockholm, Sweden; 4 Department of Health, Medicine and Caring Science, Linköping University, 581 85 Linköping, Sweden; 5 Department of Clinical Chemistry, Department of Biomedical and Clinical Sciences, Linköping University, 581 85 Linköping, Sweden; 6 Academic Healthcare Centre Stockholm, 113 65 Stockholm, Sweden

**Correction: Archives of Osteoporosis (2026) 21:46**

10.1007/s11657-026-01679-9

The correct p-value for the significant within-group increase of BMSi of 2.9% in the OsteoStrong group should be p = 0.025 and not 0.026 (which is reported in the Abstract-Results and Table 3). The correct p-value is already reported in the main text under Results.

Additionally, we also noticed that we have not reported participants who had ongoing hormone replacement therapy (HRT) at baseline. We would like to add additional information: At baseline, a total of eight participants (4.2%) were using hormone replacement therapy (OS = 4, DME = 4).

The original article has been corrected.

