# Supplementary material for: The effect of OsteoStrong compared to dynamic multicomponent exercise on bone strength in older women: the BONEMORE non-inferiority randomized controlled trial
Source: Arch Osteoporos. 2026 Feb 26;21(1):46. doi: 10.1007/s11657-026-01679-9 (PMC12946272; doi:10.1007/s11657-026-01679-9)
Supplement: Supplementary file 1 — (DOCX 16.6 KB) [file 11657_2026_1679_MOESM1_ESM.docx]

**Appendix A**

**Dynamic Multicomponent Exercise List**

All exercises were individually adapted to the participants’ physical status and capabilities.

**Session 1: Circuit exercise program** (60 minutes)
*Exercises 2-11 were performed for 40 seconds in three sets*

1. Warm-up exercises (light movements and stretches)
2. Squats
3. Side-lying hip raise
4. Supine hip raise
5. Skater jumps
6. Push-ups
7. Light jumps while walking
8. “Dead bug” (core exercise)
9. Prone back extensions
10. Step up
11. Balance exercises (e.g. one-leg standing, standing on a balance board)
12. Cool-down exercises (light movements and stretches)

**Session 2: Strength training at the gym** (60 minutes)
*Exercises 2-9 were performed 8-10 repetitions in three sets*

1. Warm-up exercises (e.g. walking on a treadmill, indoor bicycle)
2. Leg press
3. Leg curl
4. Latissimus pull-down
5. Seated row
6. Back extension (belly-back)
7. Chest press
8. Hip abduction (standing or side-lying)
9. Balance exercises
10. Cool-down exercises (light movements and stretches)
