# Supplementary material for: The effect of OsteoStrong compared to dynamic multicomponent exercise on bone strength in older women: the BONEMORE non-inferiority randomized controlled trial
Source: Arch Osteoporos. 2026 Feb 26;21(1):46. doi: 10.1007/s11657-026-01679-9 (PMC12946272; doi:10.1007/s11657-026-01679-9)
Supplement: Supplementary file 2 — (DOCX 32.2 KB) [file 11657_2026_1679_MOESM2_ESM.docx]

**Appendix B**

**Sub-analysis of Participants with and without Ongoing Bone-specific Drugs**

*It is important to note that these analyses are exploratory and should be interpreted with caution.*

**Sub-analysis of participants with bone-specific drugs – BMSi and BMD**

Age-adjusted intention-to-treat analysis within-group change, and between-group difference in BMSi, and BMD, displayed per group and time points, presented in mean ± SD. The p-values were obtained from the linear mixed model. Change (%) is calculated using the full decimal precision provided in the STATA output. P-value less then 0.05 in bold.

|  | OsteoStrong®  (n=11) | | | Within-group change | Dynamic multicomponent exercise (n=12) | | | Within-group change | Between-group difference |
| --- | --- | --- | --- | --- | --- | --- | --- | --- | --- |
|  | Baseline  Mean±SD | 9 months  Mean±SD | % Change | p-value | Baseline  Mean±SD | 9 months  Mean±SD | % Change | p-value | p-value |
| BMSi | 75.8±8.5 | 76.1±11.1 | +0.4% | 0.701 | 75.2±11.7 | 78.4±7.8 | +4.3% | 0.288 | 0.975 |
| BMD LS Total (g/cm^2^) | 0.808±0.121 | 0.815±0.131 | +0.9% | 0.554 | 0.827±0.128 | 0.817±0.121 | -1.2% | 0.09 | 0.569 |
| BMD FN Right (g/cm^2^) | 0.692±0.094 | 0.687±0.090 | -0.7% | 0.877 | 0.745±0.098 | 0.754±0.119 | +1.2% | 0.299 | 0.222 |
| BMD FN Left (g/cm^2^) | 0.667±0.049 | 0.674±0.056 | +1% | 0.693 | 0.729±0.057 | 0.712±0.052 | -2.3% | 0.386 | **0.024** |

BMSi = bone material strength index; BMD = bone mineral density; FN = femoral neck; LS = lumbar spine; SD = standard deviation.

**Sub-analysis of participants without bone-specific drugs – BMSi and BMD**

Age-adjusted intention-to-treat analysis within-group change, and between-group difference in BMSi, and BMD, displayed per group and time points, presented in mean ± SD. The p-values were obtained from the linear mixed model. Change (%) is calculated using the full decimal precision provided in the STATA output. P-value less then 0.05 in bold.

|  | OsteoStrong®  (n=86) | | | Within-group change | Dynamic multicomponent exercise (n=85) | | | Within-group change | Between-group difference |
| --- | --- | --- | --- | --- | --- | --- | --- | --- | --- |
|  | Baseline  Mean±SD | 9 months  Mean±SD | % Change | p-value | Baseline  Mean±SD | 9 months  Mean±SD | % Change | p-value | p-value |
| BMSi | 73.7±9.6 | 76.0±9.3 | +3.2% | **0.023** | 74.7±9.4 | 75.5±9.0 | +1.2% | 0.398 | 0.952 |
| BMD LS Total (g/cm^2^) | 0.882±0.130 | 0.877±0.129 | -0.6% | 0.082 | 0.872±0.127 | 0.880±0.132 | +0.9% | **0.048** | 0.600 |
| BMD FN Right (g/cm^2^) | 0.765±0.100 | 0.760±0.096 | -0.6% | 0.715 | 0.766±0.094 | 0.764±0.092 | -0.2% | 0.357 | 0.818 |
| BMD FN Left (g/cm^2^) | 0.763±0.090 | 0.766±0.090 | +0.3% | 0.089 | 0.763±0.088 | 0.768±0.093 | +0.7% | 0.330 | 0.831 |

BMSi = bone material strength index; BMD = bone mineral density; FN = femoral neck; LS = lumbar spine; SD = standard deviation.

**Appendix B (continued)**

**Sub-analysis of participants with bone-specific drugs – Bone markers**

Table 4. Age-adjusted intention-to-treat analysis within-group change, and between-group difference in bone markers displayed per group and time points, presented in median (IQR) and percentage change (compared to baseline). Change (%) is calculated using the full decimal precision provided in the STATA output. The p-values were obtained from the linear mixed model. *=Significant within-group change (p-value <0.05)

|  | OsteoStrong®  (n=11) | | | Dynamic multicomponent exercise  (n=12) | | | Between-group difference |
| --- | --- | --- | --- | --- | --- | --- | --- |
|  | Baseline  Median (IQR) | 3 months  Median (IQR), % | 9 months  Median (IQR), % | Baseline  Median (IQR) | 3 months  Median (IQR), % | 9 months  Median (IQR), % | p-value |
| PINP (µg/L) | 15.81  (12-17) | 15.76  (11-26);  -0.3% | 19.9  (15-26); **+25.7%*** | 13.8  (12-22) | 14.4  (12-16); +4.8% | 15.9  (13-18);  +15.2% | 3 months: 0.704  9 months: 0.271 |
| BALP (U/L) | 10.4  (10-14) | 13.1  (9-15);  +26.7% | 11  (9-13); +6.1% | 11.2  (9-15) | 11.9  (11-14); +5.9% | 12.8  (11-15); +14.3% | 3 months: 0.991  9 months: 0.749 |
| CTX (ng/L) | 93  (20-156) | 79  (20-146);  -21.5% | 92  (39-114);  -1.1% | 90.5  (59-158) | 67  (26-130);  -26% | 96.3  (65-122); +6.4% | 3 months: 0.759  9 months: 0.865 |
| Sclerostin (pmol/L) | 23.3  (18-28) | 25.6  (22-27);  +9.8% | 26.2  (22-29); +12.4% | 32.7  (25-41) | 31  (29-42);  -4.3% | 32  (27-40);  -2.1% | 3 months: **0.005**  9 months: **0.005** |

BALP = bone alkaline phosphatase; CTX = C-terminal telopeptide of type I collagen; P1NP = N-terminal propeptide of type-1 procollagen; IQR = interquartile range.

**Sub-analysis of participants without bone-specific drugs– Bone markers**

Table 4. Age-adjusted intention-to-treat analysis within-group change, and between-group difference in bone markers displayed per group and time points, presented in median (IQR) and percentage change (compared to baseline). The p-values were obtained from the linear mixed model. Change (%) is calculated using the full decimal precision provided in the STATA output. No significant within-group- or between-group differences detected.

|  | OsteoStrong®  (n=86) | | | Dynamic multicomponent exercise  (n=85) | | | Between-group difference |
| --- | --- | --- | --- | --- | --- | --- | --- |
|  | Baseline  Median (IQR) | 3 months  Median (IQR), % | 9 months  Median (IQR), % | Baseline  Median (IQR) | 3 months  Median (IQR), % | 9 months  Median (IQR), % | p-value |
| PINP (µg/L) | 52.5  (42-65) | 46.5  (40-63);  -11.4% | 51.8  (39-65);  -1.2% | 52  (38-63) | 50.6  (40-60);  -2.7% | 49.9  (41-60);  -4.1% | 3 months: 0.802  9 months: 0.916 |
| BALP (U/L) | 21.2  (17-25) | 22  (17-26); +3.5% | 20.5  (17-26);  -3.4% | 20.3  (17-25) | 20.5  (16-25);  +1% | 20.2  (17-24);  -0.3% | 3 months: 0.355  9 months: 0.720 |
| CTX (ng/L) | 393  (279-551) | 403  (271-520); +2.5% | 388  (285-532); -1.3% | 387  (272-549) | 393  (269-510);  +1.6% | 400  (267-484); +3.4% | 3 months: 0.906  9 months: 0.935 |
| Sclerostin (pmol/L) | 26.9  (20-33) | 25.5  (21-32);  -5.2% | 26.7  (22-33);  -0.8% | 25.1  (21-32) | 25  (21-31);  -0.1% | 26.5  (23-35);  +5.7% | 3 months: 0.881  9 months: 0.460 |

BALP = bone alkaline phosphatase; CTX = C-terminal telopeptide of type I collagen; P1NP = N-terminal propeptide of type-1 procollagen; IQR = interquartile range.
