# Supplementary material for: The effect of OsteoStrong compared to dynamic multicomponent exercise on bone strength in older women: the BONEMORE non-inferiority randomized controlled trial
Source: Arch Osteoporos. 2026 Feb 26;21(1):46. doi: 10.1007/s11657-026-01679-9 (PMC12946272; doi:10.1007/s11657-026-01679-9)
Supplement: Supplementary file 3 — (DOCX 36.1 KB) [file 11657_2026_1679_MOESM3_ESM.docx]

**Appendix C**

**Subgroup Analysis Based on Consistency in Trigger Level Achievement in OsteoStrong^®^ Sessions**

We performed a per-protocol subgroup analysis on participants in OsteoStrong^®^, comparing participants who consistently reached the trigger levels on the Lower GT and Postural GT in 100% (subgroup 1) of the sessions with those who did not (subgroup 2). Dropouts were excluded from the dataset. Excluding participants with bone-specific drug treatment did not lower the observed significant improvements. The percentage change was calculated using the full decimal precision provided in the STATA output. It is important to note that these analyses are exploratory and should be interpreted with caution.

**Lower GT**

**BMSi and BMD.** Within-group difference in BMSi, and BMD, displayed per group and time points, presented in mean ± SD. The p-values were obtained from linear mixed model contrasting the follow-up value against the baseline value. P-value less then 0.05 in bold.

|  | **OsteoStrong® subgroup 1**  (n=25)  **Reached 100%**  Mean attendance rate: 96%  Participants on bone-specific drugs: n=1 (4%) | | | Within-group change | **OsteoStrong® subgroup 2**  (n=60)  **Did not reach 100%**  (range=29-98%, mean=85%)  Mean attendance rate: 93%  Participants on bone-specific drugs: n=8 (13%) | | | Within-group change | Between-group difference |
| --- | --- | --- | --- | --- | --- | --- | --- | --- | --- |
|  | Baseline  Mean±SD | 9 months  Mean±SD | % Change | p-value | Baseline  Mean±SD | 9 months  Mean±SD | % Change | p-value | p-value |
| BMSi | 75.4±11.3 | 75.7±11.3 | +0.4% | 0.865 | 73.2±8.5 | 76.3±8.8 | +4.2% | **0.011** | 0.995 |
| BMD LS Total (g/cm^2^) | 0.904±0.144 | 0.911±0.146 | +0.8% | 0.226 | 0.853±0.118 | 0.856±0.121 | +0.04% | 0.353 | **0.049** |
| BMD FN Right (g/cm^2^) | 0.737±0.06 | 0.740±0.05 | +0.4% | 0.673 | 0.757±0.112 | 0.757±0.109 | 0% | 0.852 | 0.538 |
| BMD FN Left (g/cm^2^) | 0.760±0.07 | 0.760±0.07 | -0.01% | 0.982 | 0.752±0.102 | 0.760±0.1 | +1.1% | **0.012** | 0.914 |

BMSi = bone material strength index; BMD = bone mineral density; FN = femoral neck; LS = lumbar spine; SD = standard deviation.

**Bone markers.** Within-group changes in bone markers displayed per group and time points, presented in median (IQR) and percentage change (compared to baseline). The p-values were obtained from linear mixed model contrasting the follow-up value against the baseline value. *=Within-group change (p=≤0.05), **=Between-group difference (p=≤0.05)

|  | **OsteoStrong® subgroup 1**  (n=25)  **Reached 100%**  Mean attendance rate: 96%  Participants on bone-specific drugs: n=1 (4%) | | | **OsteoStrong® subgroup 2**  (n=58)  **Did not reach 100%**  (range=29-98%, mean=85%)  Mean attendance rate: 94%  Participants on bone-specific drugs: n=8 (14%) | | |
| --- | --- | --- | --- | --- | --- | --- |
|  | Baseline  Median (IQR) | 3 months  Median (IQR), % | 9 months  Median (IQR), % | Baseline  Median (IQR) | 3 months  Median (IQR), % | 9 months  Median (IQR), % |
| PINP (µg/L) | 47.3  (39-65) | 47.8  (42-55), +1.1% | 51.7  (39-62), +9.4% | 52  (38-64) | 45.1  (34-62),  **-13.3%*** | 47.9  (36-60), -7.9% |
| BALP (U/L) | 20.2  (15-23) | 20.5  (16-26), +1.4% | 19.8  (17-26), -1.9% | 20.9  (16-25) | 20.3  (15-26), -2.5% | 20  (15-26), -4.2% |
| CTX (ng/L) | 333  (273-467) | 386  (216-569), +16.1% | 366  (298-516),  +10% | 385  (240-575) | 369  (198-468),  -4.2% | 360  (222-530),  -6.3% |
| Sclerostin (pmol/L) | 27.1**  (23-30) | 26.7  (23-32), -1.4% | 26.9  (23-33), -0.7% | 25.5**  (19-32) | 25.2  (20-29), -1.2% | 26.4  (22-31), +3.6% |

BALP = bone alkaline phosphatase; CTX = C-terminal telopeptide of type I collagen; P1NP = N-terminal propeptide of type-1 procollagen; IQR = interquartile range.

**Appendix C (continued)**

**Postural GT**

**BMSi and BMD.** Within-group difference in BMSi, and BMD, displayed per group and time points, presented in mean ± SD. The p-values were obtained from linear mixed model contrasting the follow-up value against the baseline value. P-value less then 0.05 in bold.

|  | **OsteoStrong® subgroup 1**  (n=49)  **Reached 100%**  Mean attendance rate: 96%  Participants on bone-specific drugs: n=6 (12%) | | | Within-group change | **OsteoStrong® subgroup 2**  (n=36)  **Did not reach 100%**  (range=9-98%, mean=80%)  Mean attendance rate: 92%  Participants on bone-specific drugs: n=3 (9%) | | | Within-group change | Between-group difference |
| --- | --- | --- | --- | --- | --- | --- | --- | --- | --- |
|  | Baseline  Mean±SD | 9 months  Mean±SD | % Change | p-value | Baseline  Mean±SD | 9 months  Mean±SD | % Change | p-value | p-value |
| BMSi | 74.0±10 | 75.5±9.5 | +2% | 0.269 | 73.7±8.7 | 77.0±9.6 | +4.7% | **0.034** | 0.546 |
| BMD LS Total (g/cm^2^) | 0.884±0.128 | 0.891±0.132 | +0.8% | 0.076 | 0.853±0.125 | 0.848±0.128 | -0.5% | 0.878 | 0.131 |
| BMD FN Right (g/cm^2^) | 0.742±0.1 | 0.745±0.09 | +0.5% | 0.534 | 0.764±0.1 | 0.762±0.103 | -0.3% | 0.893 | 0.444 |
| BMD FN Left (g/cm^2^) | 0.754±0.093 | 0.760±0.096 | +0.7% | 0.163 | 0.753±0.093 | 0.760±0.086 | +0.7% | 0.132 | 0.984 |

BMSi = bone material strength index; BMD = bone mineral density; FN = femoral neck; LS = lumbar spine; SD = standard deviation.

**Bone markers.** Within-group difference in bone markers displayed per group and time points, presented in median (IQR) and percentage change (compared to baseline). The p-values were obtained from linear mixed model contrasting the follow-up value against the baseline value. No significant within-group changes were found. ******=Between-group difference (p=≤0.05)

|  | OsteoStrong® subgroup 1  (n=50)  **Reached 100%**  Mean attendance rate: 96%  Participants on bone-specific drugs: n=6 (12%) | | | OsteoStrong® subgroup 2  (n=37)  **Did not reach 100%**  (range=9-98%, mean=80%)  Mean attendance rate: 92%  Participants on bone-specific drugs: n=3 (9%) | | |
| --- | --- | --- | --- | --- | --- | --- |
|  | Baseline  Median (IQR) | 3 months  Median (IQR), % | 9 months  Median (IQR), % | Baseline  Median (IQR) | 3 months  Median (IQR), % | 9 months  Median (IQR), % |
| PINP (µg/L) | 52.262  (38-64) | 44.892  (37-60),  -14.1% | 51.701  (37-59),  -1.1% | 45.3  (39-66) | 45.1  (36-61),  +0.4% | 47.9  (37-66),  +5.7% |
| BALP (U/L) | 21.2  (17-25) | 21.5  (16-26),  +1.4% | 20.5  (17-26),  -3.1% | 17.9  (15-24) | 19.1  (15-27),  +7.1% | 19.3  (15-26),  +8% |
| CTX (ng/L) | 350  (260-575) | 375  (183-480),  +7.1% | 340  (253-516),  -4.2% | 384  (254-486) | 369  (242-469),  -4% | 379  (261-532),  -1.3% |
| Sclerostin (pmol/L) | 27.5**  (22-30) | 25.7  (22-32),  -6.7% | 27.1  (22-33),  -1.5% | 25**  (19-32) | 24.9  (20-29),  -0.7% | 24.2  (23-30),  -3.4% |

BALP = bone alkaline phosphatase; CTX = C-terminal telopeptide of type I collagen; P1NP = N-terminal propeptide of type-1 procollagen; IQR = interquartile range.
