# Supplementary material for: The effect of OsteoStrong compared to dynamic multicomponent exercise on bone strength in older women: the BONEMORE non-inferiority randomized controlled trial
Source: Arch Osteoporos. 2026 Feb 26;21(1):46. doi: 10.1007/s11657-026-01679-9 (PMC12946272; doi:10.1007/s11657-026-01679-9)
Supplement: Supplementary file 4 — (DOCX 481 KB) [file 11657_2026_1679_MOESM4_ESM.docx]

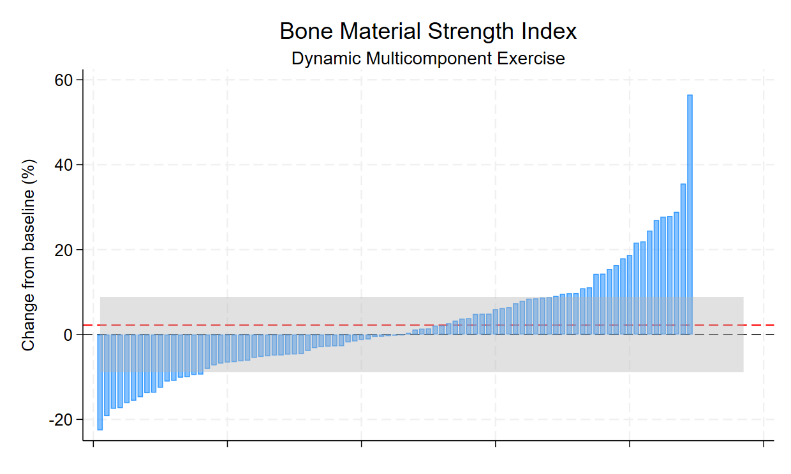

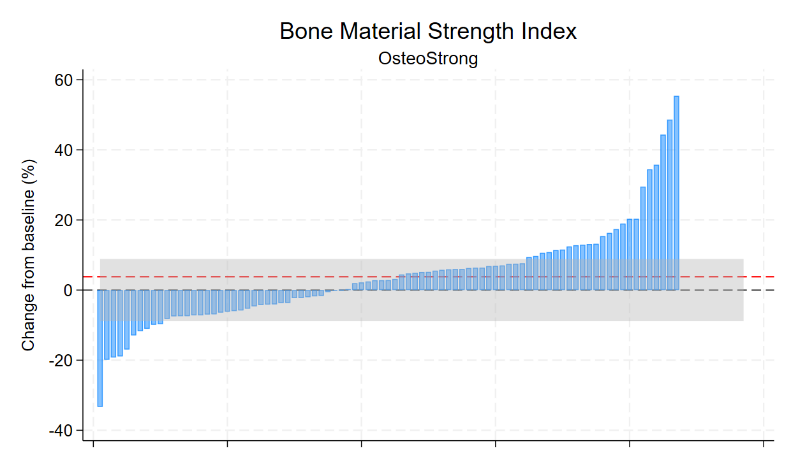

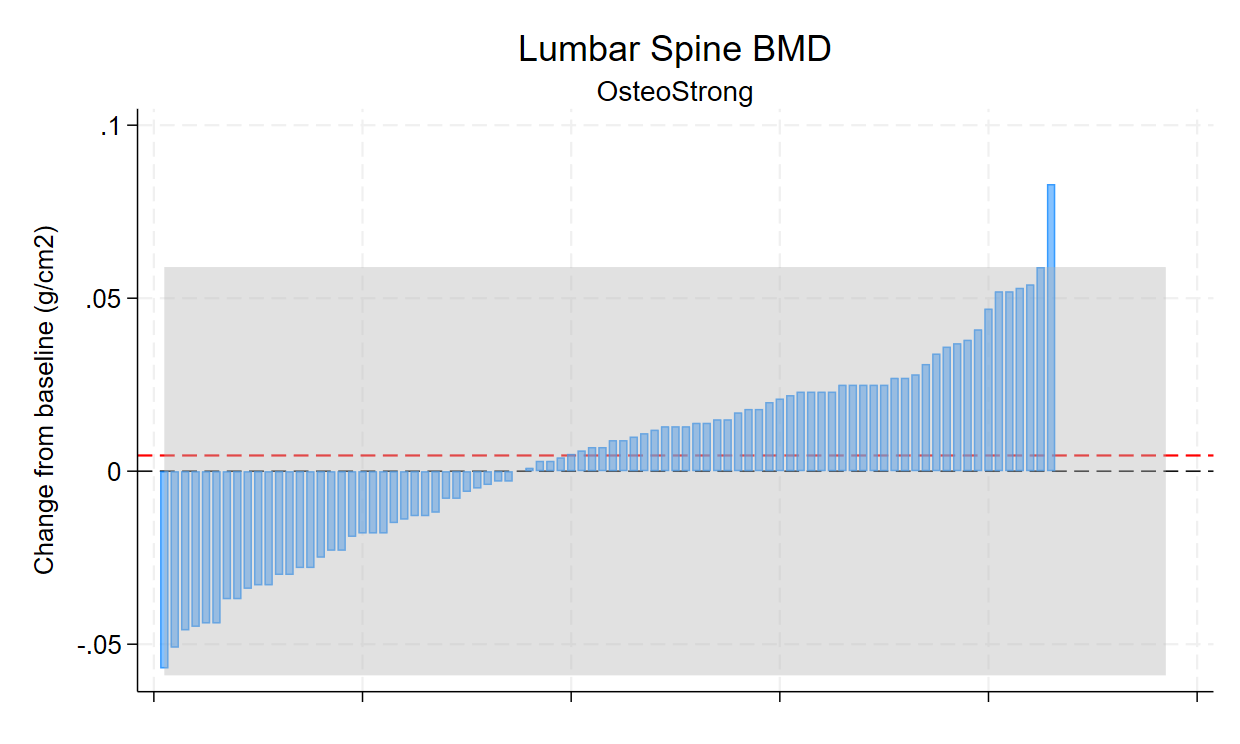

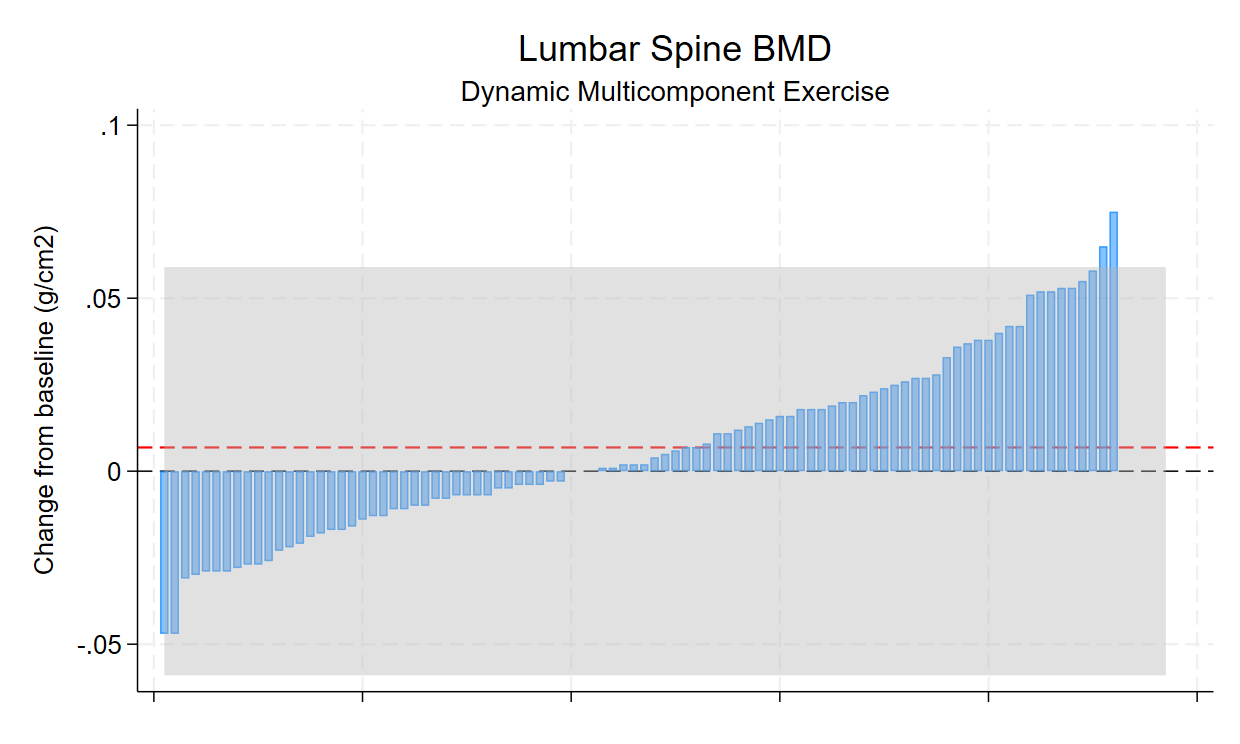

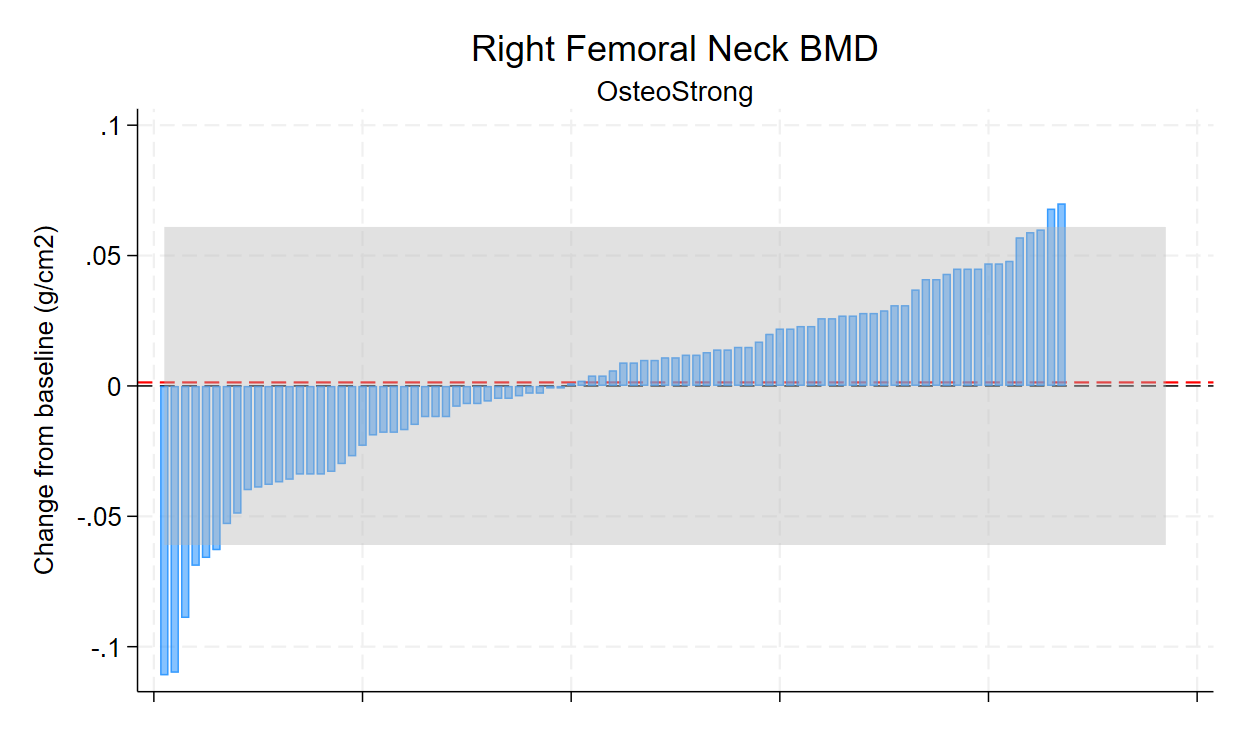

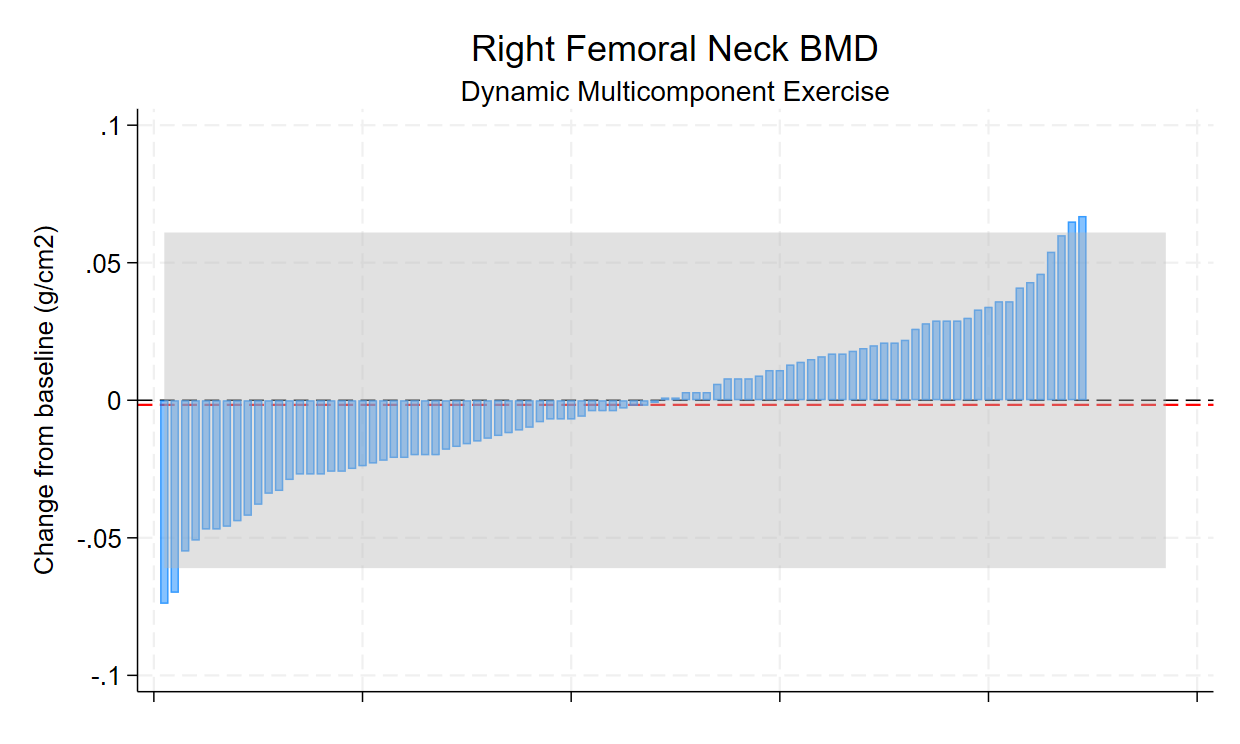

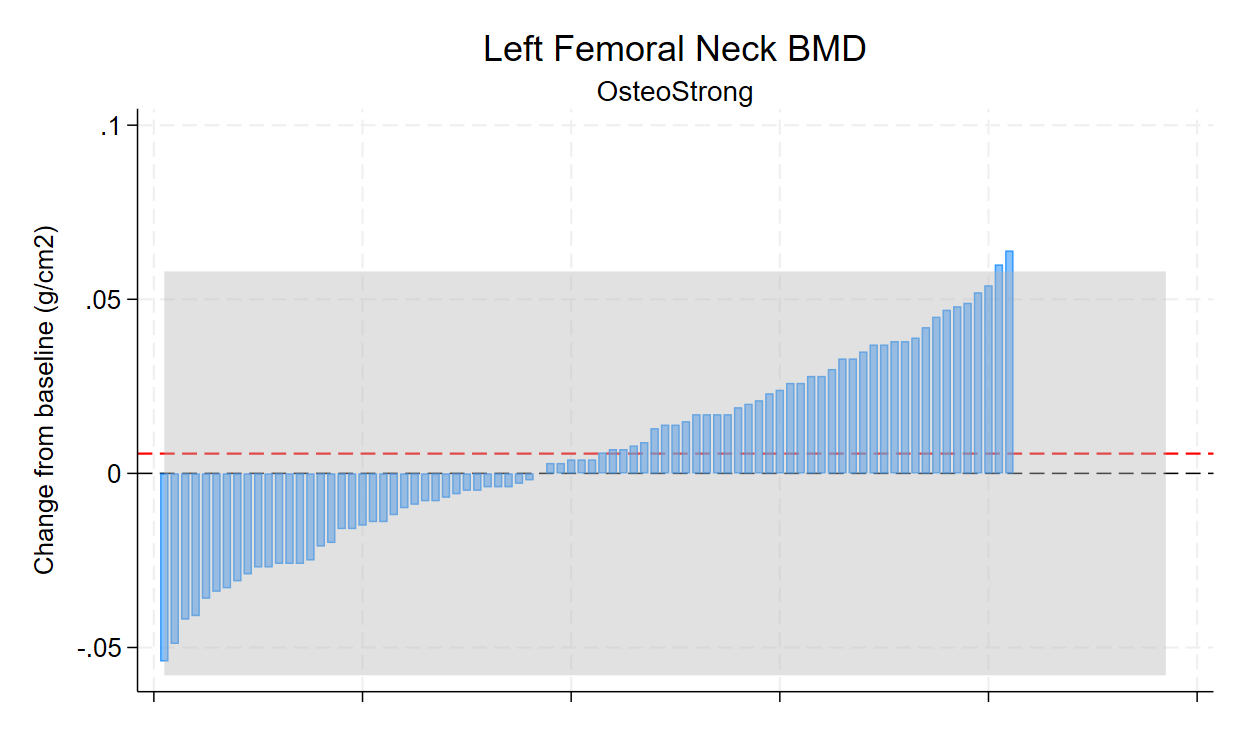

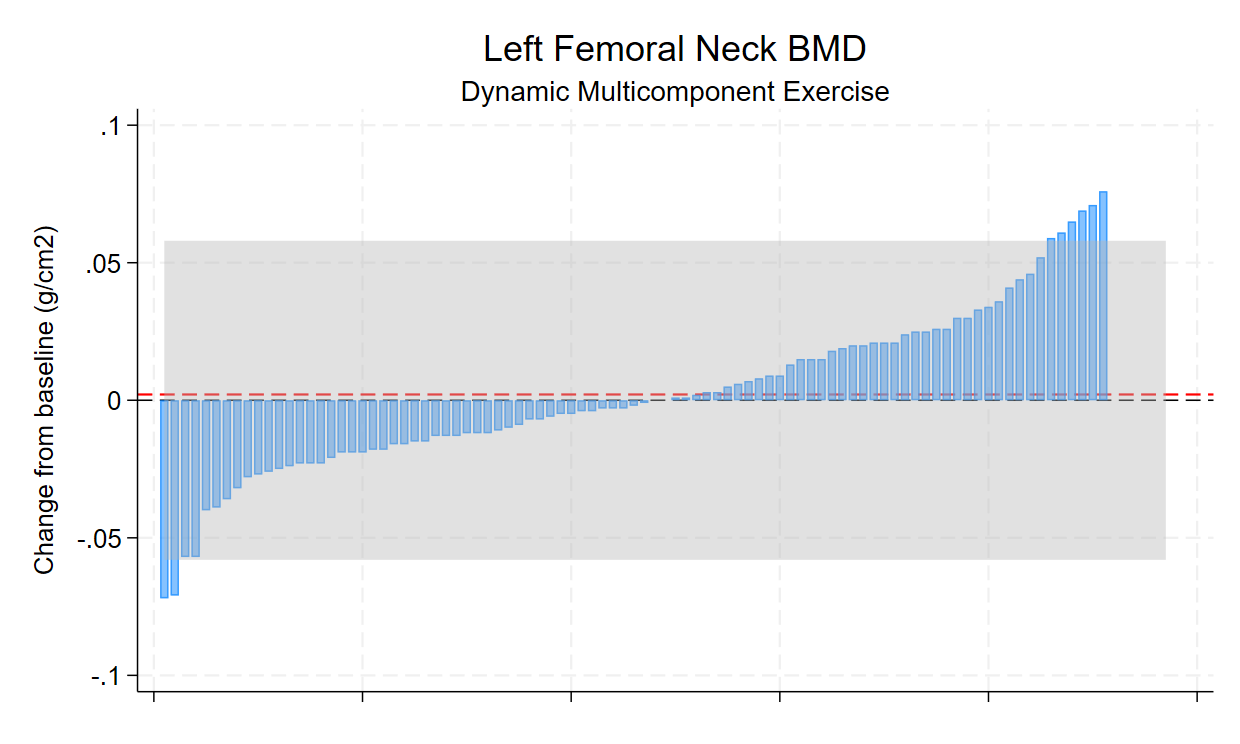


Supplementary Figure S1. Individual changes in BMSi, lumbar spine BMD, and femoral neck BMD (blue bars) and group mean (red dashed line) relative to the least significant change (shaded grey) at nine months.
