# Supplementary material for: The effect of OsteoStrong compared to dynamic multicomponent exercise on bone strength in older women: the BONEMORE non-inferiority randomized controlled trial
Source: Arch Osteoporos. 2026 Feb 26;21(1):46. doi: 10.1007/s11657-026-01679-9 (PMC12946272; doi:10.1007/s11657-026-01679-9)
Supplement: Supplementary file 5 — (DOCX 16.3 KB) [file 11657_2026_1679_MOESM5_ESM.docx]

**Supplementary Table 1**

**Participants with ongoing bone-specific drugs**

Total participants with ongoing bone-specific drugs since at least one year back = 23.

|  | OsteoStrong® | Dynamic multicomponent exercise |
| --- | --- | --- |
| Alendronate  (n, %) | 6  (55%) | 5  (42%) |
| Zolendronic acid  (n, %) | 4  (36%) | 4  (33%) |
| Denosumab  (n, %) | 1  (9%) | 3  (25%) |
| Total  (n, %) | 11  (100%) | 12  (100%) |
