# Supplementary material for: The effect of OsteoStrong compared to dynamic multicomponent exercise on bone strength in older women: the BONEMORE non-inferiority randomized controlled trial
Source: Arch Osteoporos. 2026 Feb 26;21(1):46. doi: 10.1007/s11657-026-01679-9 (PMC12946272; doi:10.1007/s11657-026-01679-9)
Supplement: Supplementary file 6 — (DOCX 44.4 KB) [file 11657_2026_1679_MOESM6_ESM.docx]

**Supplementary Tables 2-3**

**Per protocol analysis**

Supplementary Table 2. Age-adjusted per-protocol analysis within-group change, and between-group difference in BMSi, and BMD, displayed per group and time points, presented in mean ± SD. The p-values were obtained from the linear mixed model. **^a^**Within-group change (p<0.05).

|  | OsteoStrong®  (n=84) | | | Within-group change | Dynamic multicomponent exercise  (n=65) | | | Within-group change | Between-group difference |
| --- | --- | --- | --- | --- | --- | --- | --- | --- | --- |
|  | Baseline  Mean±SD | 9 months  Mean±SD | % Change | p-value | Baseline  Mean±SD | 9 months  Mean±SD | % Change | p-value | p-value |
| BMSi | 73.9±9.5 | 76.1±9.6 | +3% | **0.027** | 74.3±9.3 | 76.4±9.4 | +2.8% | 0.063 | 0.701 |
| BMD LS Total (g/cm^2^) | 0.868±0.13 | 0.873±0.13 | +0.5% | 0.117 | 0.861±0.12 | 0.863±0.13 | +0.3% | 0.444 | 0.503 |
| BMD FN Right (g/cm^2^) | 0.751±0.1 | 0.752±0.1 | +0.1% | 0.664 | 0.762±0.09 | 0.763±0.09 | +0.1% | 0.814 | 0.514 |
| BMD FN Left (g/cm^2^) | 0.754±0.09 | 0.76±0.09 | +0.8% | 0.052 | 0.751±0.08 | 0.753±0.08 | +0.3% | 0.525 | 0.631 |

BMSi = bone material strength index; BMD = bone mineral density; FN = femoral neck; LS = lumbar spine; SD = standard deviation.

Supplementary Table 3. Age-adjusted per-protocol analysis within-group change, and between-group difference in bone markers displayed per group and time points, presented in median (IQR) and percentage change (compared to baseline). The p-values were obtained from the linear mixed model. No significant differences were found within or between the groups in any of the bone markers at any time points.

|  | OsteoStrong®  (n=84) | | | Dynamic multicomponent exercise  (n=65) | | | Between-group difference |
| --- | --- | --- | --- | --- | --- | --- | --- |
|  | Baseline  Median (IQR) | 3 months  Median (IQR), % | 9 months  Median (IQR), % | Baseline  Median (IQR) | 3 months  Median (IQR), % | 9 months  Median (IQR), % | p-value |
| PINP (µg/L) | 48.9 (39-65) | 45.1 (36-61)  -8.4%  **P=0.041** | 49.5 (37-61) +1.2%  P=0.358 | 49.9 (35-61) | 47.9 (39-58)  -4.2%  P=0.753 | 47.6 (38-60)  -4.2%  P=0.406 | 3 mo: 0.844  9 mo: 0.686 |
| BALP (U/L) | 20.9 (16-25) | 20.4 (15-26)  -2.5%  P=0.370 | 20 (15-26)  -4.5%  P=0.944 | 18.9 (16-22) | 19.3 (15-23)  +2.1%  P=0.614 | 19.5 (15-23) +3.2%  P=0.225 | 3 mo: 0.841  9 mo: 0.378 |
| CTX (ng/L) | 370 (257-527) | 372 (198-480) +0.5%  P=0.307 | 366 (253-525)  -1.1%  P=0.345 | 373 (239-505) | 370 (242-508)  -0.8%  P=0.703 | 362 (241-476)  -3%  P=0.216 | 3 mo: 0.983  9 mo: 0.641 |
| Sclerostin (pmol/L) | 26.1 (20-32) | 25.5 (21-31)  -2.4%  P=0.751 | 26.5 (22-31) +1.5%  P=0.237 | 27.1 (21-33) | 25.6 (21-32)  -5.9%  P=0.353 | 27 (24-35)  -0.4%  P=0.629 | 3 mo: 0.291  9 mo: 0.181 |

BALP = bone alkaline phosphatase; CTX = C-terminal telopeptide of type I collagen; P1NP = N-terminal propeptide of type-1 procollagen; IQR = interquartile range.
